# A Cohort Study on Diabetic Undocumented Migrants in Italy: Can Charitable Organizations Contribute to Higher Adherence?

**DOI:** 10.3390/ijerph20042794

**Published:** 2023-02-04

**Authors:** Elisabetta Listorti, Aleksandra Torbica, Silvano G. Cella, Gianfrancesco Fiorini, Giovanni Corrao, Matteo Franchi

**Affiliations:** 1 Centre for Healthcare and Social Care Management (CERGAS), SDA Bocconi School of Management, Bocconi University, 20136 Milan, Italy; 2 Laboratory of Pharmacology and Pharmacoepidemiology, Department of Clinical Sciences and Community Health, University of Milan, Via Vanvitelli 32, 20129 Milan, Italy; 3 National Centre for Healthcare Research and Pharmacoepidemiology, University of Milano-Bicocca, 20126 Milan, Italy; 4 Istituti Clinici Zucchi Spa, Medicine, 20900 Monza, Italy; 5 Unit of Biostatistics, Epidemiology and Public Health, Department of Statistics and Quantitative Methods, University of Milano-Bicocca, 20126 Milan, Italy

**Keywords:** undocumented migrants, migrants, adherence, diabetes, charitable organizations

## Abstract

The increasing presence of documented and undocumented migrants increases the commitment of the Italian National Health Service to their health needs, following its founding principle of equity. In particular, chronic diseases, such as diabetes, represent a crucial area where patients’ health is affected by their adherence to care pathways, for which the recent literature has reported alarming low levels. In the case of migrants, obstacles to adherence, such as language or organizational barriers, could be overcome thanks also to charitable organizations providing healthcare services. In this study, we aimed to compare the adherence among documented and undocumented migrants who received healthcare services in Milan, Italy, either from the National Health Service (NHS) or from a charitable organization. We identified a cohort of newly taken into care diabetic patients composed of two groups: (i) documented migrants that attend the NHS; and (ii) undocumented migrants that attend a charity. Information was tracked by merging two datasets: the regional healthcare information system of Lombardy, and a unique dataset that collects data on specialistic visits and pharmaceutical prescriptions for all people visiting one of the most prominent charitable organizations in Italy. The annual diabetologist visit was used as the measure of adherence. The probability of being adherent was compared among the two groups by using a multivariate log-binomial regression model, considering a set of personal characteristics that may impact health behaviors. The cohort comprised 6429 subjects. The percentage of adherence was 52% among the documented migrants, and 74% among the undocumented. Regression results confirmed this pattern: undocumented patients have an increased probability of being adherent by 1.19 times (95% CI: 1.12 to 1.26) compared to documented ones. Our study revealed the potentiality of charitable organizations in guaranteeing continuity of care to undocumented migrants. We argue that this mechanism would benefit from central coordination by the government.

## 1. Introduction

The population trends of the last decades reveal a widespread phenomenon of migration that concerns all countries worldwide, with the number of international migrants estimated to be almost 281 million globally in 2020 [1]. Within the Italian context, around 10% of the population is immigrant, 8% of which is undocumented [2]. The increasing presence of documented and undocumented migrants raises the need for the commitment of the national healthcare systems to ensure equity in health [3]. In Italy, the National Health Service (NHS) adopts a universalistic approach by guaranteeing the same services to all its resident population, thus both to Italians and documented migrants, and the Legislative Decree 286/98 [4] clarifies for undocumented migrants the right to urgent care, essential care, preventive care diagnosis and treatment of potentially dangerous infectious diseases [5]. Nevertheless, the utilization of healthcare services by migrants is still controversial. Multiple factors play a role, among which are: (i) the lack of information about their rights to access medical services; (ii) practical obstacles such as discrimination, language, and cultural barriers; and (iii) if undocumented, the fear of being discovered and deported [6,7,8]. The topic has been addressed within several clinical areas, such as assistance during pregnancy and in the vast area of chronic diseases [9,10].

The last decade has seen an exponential increase in the role played by chronic diseases within the healthcare context. Among them, diabetes has received great attention due to several significant aspects, primarily related to its magnitude and impact: the last IDF Diabetes Atlas estimates diabetes in 2021 to be responsible for 537 million diagnoses (people aged 20–79 years), 6.7 million deaths, and a total health expenditure of at least 966 billion dollars [11]. In Italy, around 5% of the population suffers from diabetes [12], and the distribution of prevalence appears positively associated with economic and social disadvantage [13]. Moreover, an Italian group of researchers has examined ethnic variations in the prevalence of type 2 diabetes [14], which calls for targeted early intervention programs and diabetes management for high-risk groups [15].

Regarding the assistance required by diabetic patients, the concept that is vital for their health is adherence. The World Health Organization defines adherence as “the extent to which a person’s behaviour—taking medication, following a diet, and/or executing lifestyle changes—corresponds with agreed recommendations from a health care provider” [16]. Even if it is more often mentioned both in research and clinical practice as therapeutical adherence (i.e., the correct dose and timing of the pharmaceutical treatments), the concept is required to be extended to adherence to care pathways, e.g., specialistic visits or diagnostic exams that need to be undertaken with appropriate frequency. At the Italian level, the Italian Society of Diabetes has established official guidelines that, following the recent evidence [17], clarify what is meant to be adherence for a person diagnosed with diabetes [18]. After the diagnosis, patients soon receive a certificate that allows them to receive all the needed healthcare services with exemption from paying. From that point on, diabetic patients engage in a personalized care pathway, where official guidelines specify that all patients should carry out an overall visit to the diabetic facility once a year (in case the therapeutic goal is achieved and stable and there are no severe complications).

Nonetheless, several concerns arise about the low levels of adherence among diabetic patients [19,20]. Several studies have found that belonging to an ethnic minority negatively affect adherence [19,21], thus attention should be paid to the migrant population. Research performed in 2011–2015 in Tuscany revealed that compliance to the main process quality indicators is less likely by about 15–20% among migrants than non-migrants [22]. Furthermore, a higher probability of inappropriate hospital events for migrants has been observed [23,24,25]. Most of the studies that have explored the dynamics underlying the phenomenon have focused on documented rather than undocumented migrants [26,27]. The population of undocumented migrants has peculiar characteristics, many of which cannot be explicitly documented due to the lack of data caused by the difficulty in tracking and following them for extended periods. Nonetheless, the phenomenon described so far can be reasonably thought of as emphasized in the case of unregular immigration due to the fear of not owning the right to health.

In this panorama, a strategic role could be played by charitable organizations, which have been recognized as having the potential to provide health care services at prevention, treatment, and rehabilitation levels [28]. In Italy, like in other European countries, there is a widespread net of nongovernmental organizations (NGOs) that provide healthcare services to people in need, and that are mainly run voluntarily [29,30]. Charities have essential roles in the healthcare sector, such as addressing socioeconomic disparities resulting in poor health and outcomes. They do so mainly by providing care to all people knocking at their doors and trustworthy information resources for patients and families [31]. The potential beneficial effects of this behaviour have been shown in previous studies, documenting that the utilization of health care services by migrants may be effectively increased by the implementation of a series of migrant-oriented practices [8].

All this considered, in this paper, we investigated whether migrant populations are managed differently by the NHS (documented migrants) or charitable organizations (undocumented migrants). Our findings explored whether charities could represent a strategical gatekeeper of the healthcare system for undocumented migrants, contributing to setting the steppingstone that allows them access to the universalistic NHS. We approached this issue by focusing on the concept of adherence in diabetes. In order to deepen this point, we identified a cohort of newly taken into care diabetic patients of foreign citizenship composed of two groups, which differ because they include (i) documented migrants that receive healthcare services from the NHS; and (ii) undocumented migrants that make use of healthcare services within a charitable organization. More specifically, we aimed to evaluate which factors affect adherence, intended as receiving at least one diabetologist visit during the year following the inclusion into the study. In particular, among the factors considered, we aimed to highlight the difference in adherence that exists among migrants that attend NHS services and those who do not. We focused the discussion on the possible explanations of such dynamics. Results will help to shed some light on the integration mechanisms that may enable migrants of any type to access the NHS freely.

## 2. Materials and Methods

### 2.1. Data Source and Study Design

Our study is a retrospective cohort study. The study cohort was composed of two groups of diabetic patients, all with not-Italian citizenship, whose information has been tracked by using two distinct datasets. In particular, the first group was composed of documented migrants that are resident in Milan and have access to the national health service (NHS) through the regional health service of Lombardy. We shortened this to “Documented NHS” (information coming from the first dataset). For the second group (that was shortened to “Undocumented OSF), we considered all the undocumented migrants that visited a charitable organization in Milan, i.e., Opera San Francesco (OSF).

OSF in one of Italy’s most prominent charitable organizations, which has guaranteed free assistance and shelter to the needy in Milan since 1959. OSF delivers medical assistance through outpatient clinics for almost all specialties, and the generous donations of its supporters allow medicines to be dispensed for free to patients according to prescriptions after each consultation. Moreover, OSF pursues a mapping effort by keeping track of all the information of all patients seen during the last decade. This wealth of data has enabled researchers to enlighten aspects of migrants’ health that are of strategical importance for the Italian healthcare system, such as risk factors and the epidemiology of diseases [29].

The two datasets we referred to consisted of: (1) the regional healthcare information system of Lombardy, which contains data on the healthcare services provided through the NHS to the ten million inhabitants of the region over the last few decades; and (2) a dataset that collects information on all people visiting OSF, which owns data on specialistic visits since 2009 and on pharmaceutical prescriptions from June 2011 [32].

Out of all the populations considered in the two datasets, we considered only patients with not-Italian citizenship living in Milan. Among them, for both groups, we selected diabetic patients by following the literature of case-identification algorithms based on Italian healthcare administrative databases [33]. In the group of Documented NHS, we identified diabetic patients through one out of three elements: (i) the presence of at least two prescriptions within the same year for antidiabetic medications (ATC drug code A10), and (ii) the diagnosis of diabetes from any hospital discharge record (ICD-9-CM diagnostic code 250); (iii) the exemption from medical charges for a diagnosis of diabetes (code: 013, RJ0010, 012, 012.253.5, 013.250). Adapting the same rationale to the different healthcare services organization in OSF (e.g., the absence of hospitalizations), for the Undocumented OSF, we identified diabetic patients as those subjects that had at least two prescriptions for antidiabetic medications, or a diagnosis of diabetes from any of the visits made in OSF.

**Figure 1:**
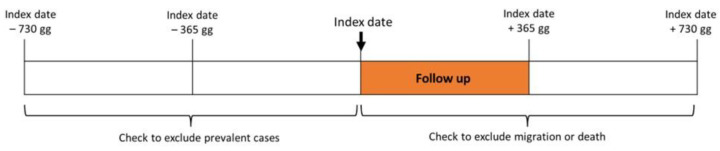
Time windows for the identification of the cohort.

Figure 1 graphically describes the methodology used for the identification of the cohort. 

The date of the first contact with either the NHS or the OSF for diabetes was labelled “index date”, i.e., the documented date of diagnosis. In order to include into the study cohort only patients newly taken into care for diabetes, we excluded patients who had either a diagnostic code of diabetes or an antidiabetic medication in the two years before the index date. Patients were followed up from the index date to the next year (one-year follow-up). To be able to map the health episodes during the one-year follow-up, we excluded from the study people that have migrated or died during the one-year of follow-up, based on the available information: for the Documented NHS, we included only those subjects that were beneficiaries of the NHS and resident in Milan during the one-year follow-up; for the Undocumented OSF, since no information on migrations or death is available in OSF databases, we included only those subjects that made at least one access of any type in OSF during the period from the end of the follow up to the following year. 

Given that (i) complete information on Undocumented OSF as for both visits and drugs is available from 2012, (ii) we decided to stop our observation period at the beginning of 2020 to avoid the consideration of biases related to COVID-19, and (iii) the enrolment process reported in Figure 1, all subjects included in the cohort have been enrolled between 2014 and 2018.

### 2.2. Variables

Other than the exposure of interest (i.e., receiving healthcare by the NHS or by OSF), we retrieved for the whole cohort the main demographic data: age, sex, and country of birth. We then recorded all pharmacological treatments and characterized patients by the type of therapy followed during the follow-up, classified into four levels: none (i.e., no ATC A10 prescriptions), oral antidiabetic drugs only (i.e., ATC A10B prescriptions), insulin (i.e., ATC A10A prescriptions), the combination of insulin and oral antidiabetic drugs (i.e., ATC A10A and ATC A10B prescriptions).

The dependent variable for the study is the indicator of adherence: a boolean variable that has value 1 if the subject has made at least one diabetologist visit during the one-year follow-up, zero otherwise. In this way, we used one of the indicators specified in the national guidelines, of which all the practitioners are well informed [17]. We tracked the visits with diabetologists either through the outpatient’s specialist visits (Regional codes 89018 or 897A8) for Documented NHS, or with the label of “diabetologist” within the information on specialistic visits performed for Undocumented OSF.

### 2.3. Statistical Analysis

We performed a set of descriptive statistics to report the main characteristics of our cohort and the existing differences in adherence levels as depending on our set of independent variables, which we outlined graphically. We ultimately ran a log-binomial regression to enlighten the direction and magnitude of the association of the independent variables on the probability of being adherent. Stratified analyses by sex were also performed. Estimates were reported as risk ratios (RR), along with 95% confidence intervals (CI). An additional check of the results was made by performing an analysis with the use of the Propensity Score Matching between Documented NHS and Undocumented OSF, based on the same independent variables used for the main analysis. The results from the log-binomial regression performed on the matched dataset confirmed the main results, thus are not shown in the following, although available upon request. All analyses were performed with R and SAS.

## 3. Results

The cohort is composed by 6429 subjects, of which 274 belong to the group of Undocumented OSF and 6155 to the group of Documented NHS. The number of subjects enrolled by year varies between 44 and 73 (Undocumented OSF) and between 1152 and 1280 (Documented NHS). 

Table 1 reports the characteristics of the two groups, revealing some differences among them, such as their continent of origin and their pharmaceutical treatment. While almost half (43%) of the Documented NHS come from the Asian continent, the most frequent continent of origin of the Undocumented OSF is America (42%). Another difference was in the percentage of patients having no pharmaceutical treatment, which was 26% in the Documented NHS group and 12% in the Undocumented OSF group. Most importantly, the level of adherence differentiates the two groups: 52% of Documented NHS had been visited by a diabetologist during the year following the diagnosis, while this percentage rose to 74% for the Undocumented OSF.

**Figure 2:**
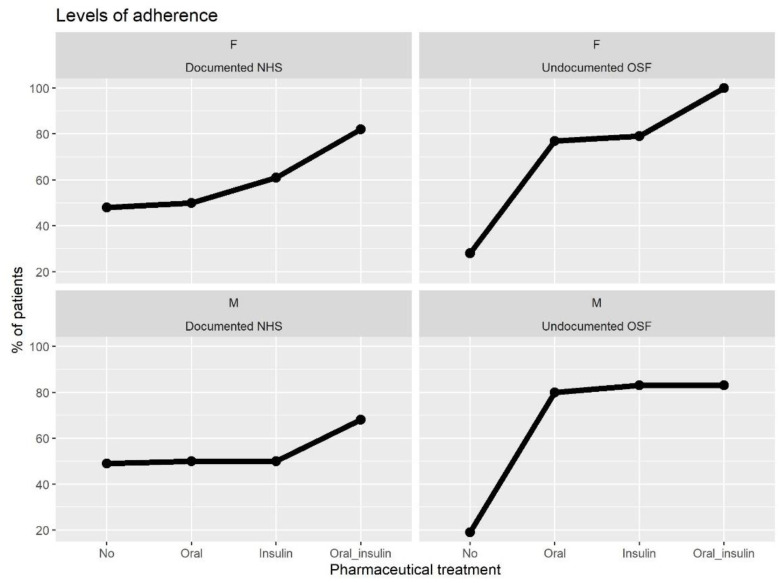
Adherence by sex and therapy, divided for the groups Undocumented OSF and Documented NHS.

**Figure A1:**
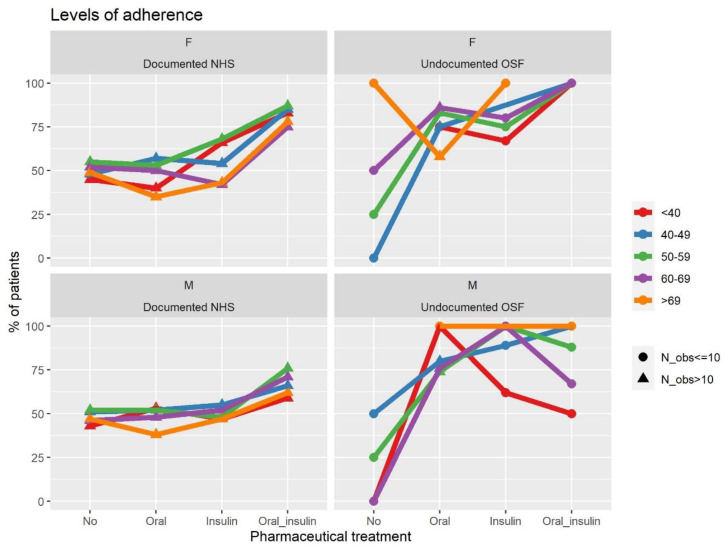
Adherence by sex, age class and therapy, divided for the groups Undocumented OSF and Documented NHS.

Figure 2 deepens this difference by observing the adherence levels of the population characterized in terms of sex and therapy. For both groups, different pharmaceutical treatments correspond to rather different adherence levels. In particular, a gradient that associates more complex therapeutical treatments to higher adherence seems to exist, which is recurrent for Undocumented OSF vs. Documented NHS, and for male as for female. Adherence levels for patients not subjected to pharmaceutical treatment are less than 30% and appear to be very similar in the Undocumented OSF and Documented NHS groups. However, the association between adherence and complexity of the therapy appears more pronounced for Undocumented OSF patients. Figure A1 provided in the Appendix A deepens the same variables in relation also to age. Even if it seems that younger patients are more adherent than older, the trend is not straightforward. Eventually, few categories of Documented NHS appear to be more adherent than their corresponding Undocumented OSF.

Even if the adherence seems confirmed to be higher for the Undocumented OSF, the presence of multiple differences among the groups calls for a regression to observe the adherence levels ceteris paribus.

Regression results in Table 2 enrich the main points that have emerged so far. The main result of our analysis is the direction and magnitude of the variable identifying the Undocumented OSF vs. Documented NHS: all considered, being a patient of the Undocumented OSF group increases the probability of being adherent of 1.19 times (95% confidence interval: 1.12 to 1.26) compared to the Documented NHS group. Corresponding figures from the stratified analyses among men and women were 1.38 (1.24 to 1.54) and 1.11 (1.03 to 1.21), respectively (*p* = 0.002). Being aged over 69 years was associated to a reduction in the probability of being adherent of 8% (2% to 15%), with respect to those aged less than 40 years. As compared to people coming from Africa, those coming from America, Asia and Europe were associated to an increased probability of being adherent of 5% (0% to 10%), 5% (1% to 9%) and 6% (1% to 12%), respectively. Finally, significant results come out for the role played by the pharmaceutical treatment. The higher the complexity of the treatment, the higher the probability of being adherent. In particular, patients treated with both oral and insulin therapy were associated to an increased probability of being adherent of 31% (24% to 37%), as compared to those untreated.

## 4. Discussion

The provision of healthcare services to immigrants is a crucial issue, especially when focusing on the need for chronic patients of adherence and continuity of care. The risk of inappropriateness, which is a significant theme in terms of healthcare expenditure, emerges alongside the risk of not accessing the NHS. The literature has shown that the immigration status of an individual is a major factor that negatively affects that person’s ability to seek and experience healthcare services [7,34]. Undocumented migrants, compared to documented ones, face further challenges, such as the fear that seeking health care would result in their being reported to the authorities. Overall, studies have shown an underutilization of healthcare services by undocumented migrants [35]. This point makes it necessary to identify specific strategies to reach undocumented migrants with healthcare services [36].

There are mainly two streams of access to healthcare services for undocumented migrants, i.e., conventional healthcare facilities and informal systems, such as charitable organizations. On one side, several previous studies, and also a recent Italian work focusing on diabetic undocumented migrants, have reported different usage of conventional healthcare services, represented by more urgent hospital admissions, more preventable complications, and a higher recurrence in terms of access and costs to hospital services rather than drugs [37]. On the other side, as stated in a recent editorial, many charities provide impactful direct care provision as well as trustworthy information resources for patients and families of paramount importance. Hence, even though it can be challenging to measure the direct impact that the voluntary sector has on healthcare systems because of the complexity of these systems, there is the need to recognize the critical roles played by charities, especially in addressing socioeconomic disparities that may otherwise result in poor health and outcomes [31].

Following this need, our study documents that, taking into account personal characteristics that may impact on healthcare behaviours (such as age, sex, nationality and pharmacological treatment), attending a charitable organization as OSF, compared to receiving healthcare services provided by the NHS, increases the probability of being adherent to the diabetologist visit prescribed annually to diabetic patients. Anecdotal evidence collected during our work suggests that what enables charities to reach this achievement is their capacity to reach undocumented immigrants with a closer and person-oriented approach that creates a strong bond between the patient and OSF—which are defined as “migrant-oriented practices”. OSF offers a holistic service composed of general practitioners, drug delivery and specialistic visits, and cultural mediators and interpreters that enable overcoming linguistic barriers. Moreover, people attending OSF may receive not only medical services but other services, such as food, social cohesion, and leisure, which contribute to decreasing their opportunity cost of attending, and is different to what happens with NHS healthcare facilities. Similar features have also been reported by another previous Italian work that documented the higher propensity of undocumented migrants to use the services offered by volunteers [8].

Our study supports reflections on health policies directed to the care of undocumented migrants, which recall previous findings on the potential role of the NHS to tackle inequities in health [38]. With undocumented migrants, though, the ability of the NHS to treat patients must be enriched by the capacity to reach them. As encouraged in a recent Italian work [27], it is necessary to adopt strategies of system mediation that make healthcare services more sensitive to cultural specificities. At the Italian level, there are significant experiences of “proximity medicine” on vulnerable groups, such as the one studied in this research, which support the picture of charitable organizations as tools that the NHS could use to offer healthcare services to undocumented migrants and guarantee the continuity of care. However, the stakeholders involved in charities would benefit from central coordination by the government, taking more operational steps towards supporting such organizations, e.g., by granting special facilities and exemptions, engaging charities in policymaking and training processes, and empowering them in terms of the production of resources [28]. If correctly managed by the central level, the net of NGOs and charitable organizations could fulfil that significant potential for developing culturally and linguistically appropriate, clinically sound, and cost-effective interventions that is advocated [39] to respond to the growing migrant and ethnic minority populations affected by diabetes worldwide.

Our study presents strengths and limitations, mostly coming from the data used. In fact, the main characteristic of our study is that of an observational study with a retrospective approach. Consequently, we do not have the possibility of choosing the people included in the study, nor their number. This fact resulted in the sample size of Undocumented OSF being smaller than the one of Documented NHS. However, this aspect comes with all the advantages of a real-world-based study. Another challenge is represented by dealing with undocumented migrants’ data, which raises some issues related to the difficulty of tracking their stories. In our case, we assumed identifying newly taken into care diabetic cases among undocumented migrants through their first contact for diabetes received in OSF. However, those patients could have moved from other countries/cities where they had already received a diagnosis, thus being prevalent cases with different adherence characteristics. Despite this limitation, OSF provides a unique dataset with information on health services used by undocumented migrants for a considerable amount of time. This characteristic, which represents a great value added given the difficulty that research on migration faces with data availability [40], enabled us to report a comparison of the two healthcare systems, i.e., NHS and OSF. Moreover, considering an indicator of adherence that refers to the care pathway opens the discussion to a broader view of the treatment of chronic diseases, which departs from only pharmacological treatments. Overall, we believe that the approach of a real-world-based study with the inclusion of rich information on undocumented migrants, supports the development of evidence-informed health interventions directed at migrants.

## 5. Conclusions

In this paper, we addressed the crucial topic of adherence among diabetic migrant patients, focusing on undocumented migrants. Our results show that taking into account personal characteristics that may impact healthcare behaviours, receiving healthcare services within a charitable organization rather than from the NHS increases the probability of being adherent by 1.19 times (95% CI: 1.12 to 1.26). On one side, this result echoes with previous studies that have encouraged to value the migrant-oriented practices adopted by charities, which make healthcare services more sensitive to cultural specificities. On the other side, this result enables a quantification of the potential impact of charitable organizations, and thus strengthens the need for central coordination by the government.

## Figures and Tables

**Table 1:** Characteristics of the cohort.

		Documented NHS *n* = 6155	Undocumented OSF *n* = 274	*p*-Value Chi-Squared Test
**Sex**	**F**	55% (3368)	48% (131)	0.03
	**M**	45% (2787)	52% (143)	
**Age**	**<40**	21% (1306)	12% (32)	*p* < 0.01
	**40–49**	23% (1441)	26% (71)	
	**49 < age < 60 50–59**	28% (1731)	34% (92)	
	**59 < age < 70 60–69**	17% (1075)	22% (59)	
	**Age > 69 70+**	10% (602)	7% (20)	
**Continent**	**Africa**	28% (1750)	21% (56)	*p* < 0.01
	**America**	14% (846)	41% (112)	
	**Asia**	43% (2649)	11% (31)	
	**Europe**	13% (800)	27% (75)	
	**Missing**	2% (110)	0% (0)	
**Therapy**	**No**	26% (1623)	12% (34)	0.01
	**Oral**	54% (3348)	62% (169)	
	**Insulin**	10% (592)	14% (38)	
	**Oral and insulin**	10% (592)	12% (33)	

**Table 2:** Results from regression.

Independent Variable		Risk Ratio (95% Confidence Intervals)	*p*-Value
Undocumented OSF (vs. Documented NHS)		1.19 *** (1.12–1.26)	<0.001
Gender Male (vs. Female)		0.97 * (0.94–1.00)	0.072
Age class (vs. under 40 years)	40–49 years	1.03 (0.98–1.08)	0.189
	50–59 years	1.04 * (0.99–1.09)	0.100
	60–69 years	1.00 (0.95–1.05)	0.927
	Over 69 years	0.92 ** (0.85–0.98)	0.014
Continent (vs. Africa)	America	1.05 * (1.00–1.10)	0.067
	Asia	1.05 *** (1.01–1.09)	0.019
	Europe	1.06 *** (1.01–1.12)	0.032
Therapy (vs. no therapy)	Oral therapy	1.04 *** (1.00–1.09)	0.043
	Insulin therapy	1.13 *** (1.06–1.19)	<0.001
	Oral and insulin therapy	1.31 *** (1.24–1.37)	<0.001

* *p* < 0.1; ** *p*< 0.05; *** *p* < 0.01.

## Data Availability

Lombardy Region: The data that support the findings of this study are available from Lombardy Region, but restrictions apply to the availability of these data, which were used under license for the current study, and so are not publicly available. Data are however available from the Lombardy Region upon reasonable request. OSF: The data used to support the findings of this study are available on reasonable request.
